# Validation of the Non-Motor Symptoms Scale for Parkinson's Disease of Persian Version

**DOI:** 10.1155/2023/1972034

**Published:** 2023-06-09

**Authors:** Zahra Eghlidos, Aida Abolhassanbeigi, Zahra Rahimian, Samaneh Khazraei, Vahid Reza Ostovan

**Affiliations:** ^1^Student Research Committee, Shiraz University of Medical Sciences, Shiraz, Iran; ^2^Department of Psychiatry, Shiraz University of Medical Sciences, Shiraz, Iran; ^3^Clinical Neurology Research Center, Shiraz University of Medical Sciences, Shiraz, Iran; ^4^Department of Neurology, Shiraz University of Medical Sciences, Shiraz, Iran

## Abstract

**Objective:**

We aimed to assess the validity and reliability of the Persian version of the NonMotor Symptoms Scale (NMSS) in Iranian patients with PD.

**Methods:**

This cross-sectional study was conducted in patients with PD. After the cross-cultural adaptation of the NMSS, the acceptability, reliability, precision, and validity of the Persian NMSS were evaluated. For this purpose, in addition to NMSS, we used the following measures: Scales for Outcomes in Parkinson's Disease (SCOPA)-Autonomic (SCOPA-AUT), SCOPA-Sleep, Beck's Depression Inventory (BDI) questionnaire, Parkinson's Disease Questionnaire-8 questions (PDQ–8), SCOPA-Motor, SCOPA-Psychiatric Complications (SCOPA-PC), SCOPA-Cognition (SCOPA-COG), Mini-Mental State Examination (MMSE), Hoehn and Yahr Staging (H and Y), and Unified Parkinson Disease Rating Scale (UPDRS).

**Results:**

186 patients were enrolled **(**mean age 64.46 ± 9.9 years; disease duration 5.59 ± 3.99 years; 118 (63.4%) male; mean NMSS score 52.01 ± 38.54). Neither the floor effect (2.7%) nor the ceiling effect (0.5%) was seen in NMSS total score. Cronbach's alpha of total NMSS was 0.84. The test-retest reliability was 0.93 for the NMSS total and 0.81–0.96 for domains. The standard error of measurement (SEM) was lower than half of the standard deviation for NMSS total and all domains. NMSS total showed a high correlation with UPDRS I (*r*_*s*_ = 0.84), UPDRS II (*r*_*s*_ = 0.58), PDQ-8 (*r*_*s*_ = 0.61), BDI (*r*_*s*_ = 0.71), SCOPA-sleep (*r*_*s*_ = 0.60), and SCOPA AUT (*r*_*s*_ = 0.66). NMSS has an acceptable discriminative validity based on disease duration and severity of disease according to H and Y staging.

**Conclusion:**

The Persian NMSS is a valid and reliable measure for evaluating the burden of nonmotor symptoms in Iranian patients with PD.

## 1. Introduction

Parkinson's disease (PD) is a progressive neurodegenerative disease that causes both motor and nonmotor symptoms. It is characterized by dopaminergic and nondopaminergic neuron degeneration in various parts of the central nervous system, particularly in the brain stem [1, 2]. Although motor symptoms are primarily due to the loss of dopaminergic neurons, nonmotor symptoms (NMS) result from the degeneration of both nondopaminergic and dopaminergic neurons and neurotransmitter systems [3]. The cardinal motor symptoms of PD include bradykinesia, rigidity, postural instability, and resting tremor [4]. NMS can be present at all stages of the disease, with a prevalence of 21% at the time of diagnosis of PD and 90% after seven years of disease duration [3, 5]. Besides, in a large international study, less than 2 percent of patients did not report any kind of NMS [6]. NMS includes cognitive impairment, psychiatric symptoms, autonomic dysfunction, gastrointestinal symptoms, sleep disturbances, fatigue, sensory symptoms, and olfactory dysfunction. Amongst these symptoms, constipation, rapid eye movement (REM) sleep behavior disorder (RBD), depression, and olfactory symptoms may be present for years before the initiation of motor symptoms [3]. NMS considerably adversely affects the patient's quality of life and psychosocial performance [7]. NMS is relatively overlooked, and diagnosis of more than 50% of existing NMS may be missed in clinical practice [3, 8]. Thus far, only four efficient questionnaires including the Nonmotor Questionnaire (NMSQuest) [9], the Nonmotor Symptoms Scale (NMSS) [10], the Movement Disorder Society Non-Motor Rating Scale (MDS-NMS) [11], and the Movement Disorder Society Unified Parkinson Disease Rating Scale I (MDS-UPDRS I) [12] have been developed for screening and assessment of the severity and frequency of NMS. Considering the lack of a valid and reliable instrument for evaluating NMS in Iranian patients, this study aimed to assess the psychometric properties of a Persian version of the NMSS in the Iranian population with PD.

## 2. Methods

### 2.1. Study Design and Participants

This observational, cross-sectional, single-center study was conducted on consecutive patients with PD in the movement disorder clinics affiliated with Shiraz University of Medical Sciences, Shiraz, Iran. We recruited patients diagnosed with PD from April 2021 to March 2022. Diagnosis of PD was defined according to the Movement Disorder Society Clinical Diagnostic Criteria [13]. PD patients older than 40 years were included. Exclusion criteria were a history of repeated strokes or head injury with the stepwise progression of Parkinsonian features, severe dementia (minimental state examination (MMSE) score ≤10) [14], encephalitis, oculogyric crisis, neuroleptic treatment at the onset of symptoms, supranuclear gaze palsy, cerebellar signs, early severe autonomic involvement, unexplained Babinski sign, and exposure to toxic agents.

### 2.2. Standard Protocol Approvals and Patients' Consent

The study protocol was approved by the ethics committee and institutional review board of Shiraz University of Medical Sciences (Approval No# IR.sums.med.rec.1399.470). All patients provided informed consent before they participated in the study. The rating scale translation was created with permission from the NMSS copyright holder, the International Parkinson and Movement Disorder Society (MDS).

### 2.3. Assessment

Through interviews with patients, the following data were provided: age, gender, marital status, education, and occupation; age at onset, duration of disease, and pharmacological treatment (levodopa equivalent daily dose (LEDD) [15]). Functional assessment of PD patients contained neurologist-based and patient self-assessments. The assessments were carried out on patients in the “medication on” state.

#### 2.3.1. Neurologist-Based Assessment

The NMSS [10] is a scale designed to evaluate nonmotor symptoms in PD during the previous month. There are nine domains and thirty items in total as follows: cardiovascular (2 items), sleep/fatigue (4 items), mood/apathy (6 items), perceptual problems/hallucinations (3 items), attention/memory (3 items), gastrointestinal tract (3 items), urinary (3 items), sexual function (2 items), and miscellaneous (4 items). To capture symptoms that are severe but occasional or less severe but persistent, items are rated for severity (from 0 to 3) and frequency (from 1 to 4). Three hundred sixty points are the potential maximum total score. Besides, Scales for Outcomes in Parkinson's Disease (SCOPA)-Motor (SCOPA-M) (3 domains and 21 items in total, each item scoring from 0 to 3, with total score ranging from 0 to 63) [16], SCOPA-Psychiatric Complications (SCOPA-PC) (7 items, each item scoring from 0 to 3, a total score ranging from 0 to 21) [17], SCOPA-Cognition (SCOPA-COG) (5 domains 10 items, total score range from 0 to 43) [18], MMSE (5 domains, total score ranging from 0 to 30) [19], Hoehn and Yahr Staging (H and Y) [20], and UPDRS [12] are considered.

#### 2.3.2. Patient Self-Assessments

SCOPA-Autonomic (SCOPA-AUT) (26 items, total score ranging from 0 to 71) [21], SCOPA-Sleep Scale (4 domains, total score ranging from 0 to 39) [22], Beck's Depression Inventory (BDI) questionnaire (21 items, each item scoring from 0 to 3, a total score ranging from 0 to 63) [23], Parkinson's Disease Questionnaire-8 questions (PDQ-8) (8 items, total score ranging from 0 to 32) [24] were calculated.

For all rating scales except SCOPA-COG and MMSE, higher scores indicate greater severity, whereas, for SCOPA-COG and MMSE, lower scores correspond to worse cognitive performances.

### 2.4. Cross-Cultural Adaptation of the NMSS

The NMSS was cross-culturally adapted using a previously developed process for translation and back-translation [25]. The NMSS was initially translated into Persian by two independent native speakers with solid English knowledge, and then a consensus version of the NMSS was reached. Second, without access to the original English version, the consensus version was back-translated into English by an English native speaker with high proficiency in Persian. Third, the NMSS developers compared the first back-translation to the original version and made changes to eliminate any differences between the original and the back-translated version. Ultimately, the latest version was applied to 20 patients to assess the cognitive debriefing and understanding of the questions. No significant issues were aroused during cognitive pretesting; subsequently, the final version was provided, namely, NMSS-PD Persian version. These 20 patients were excluded from our study.

### 2.5. Data Analysis

To assess the stability of the Persian version of the NMSS (test-retest reliability), a group of 50 patients (*n* = 50) repeated the NMSS two to four weeks following the initial assessment with a different researcher from the one who conducted the initial evaluation.

The following characteristics were also examined for the Persian version of the NMSS:

  Data quality: If more than 95% of the NMSS data were completely computable, the proportion of computable data was deemed adequate [26].
  Acceptability: The range of scores, skewness (limits: −1 to +1), and the floor and ceiling effects (maximum acceptable value for both: 15%) were calculated [27].
  Reliability: Internal consistency of the NMSS domains was assessed using corrected item-domain correlation [28] and Cronbach's alpha coefficient [29] (values ≥ 0.30 and ≥ 0.70, respectively, were considered appropriate). The Intra-Class Correlation (ICC) was used to examine test-retest reliability in a group of 50 patients after two to four weeks, with values greater than 0.70 considered satisfactory [30].
  Precision: The standard error of measurement (SEM) was used to determine precision for the NMSS domains; the less the standard error of measurement, the more trustworthy the test (an SEM value of less than half of the standard deviation was used as a criterion of acceptable precision) [29].
  Validity: We used the Spearman rank correlation coefficient (*r*_(*s*)_) to investigate the relationship between the different domains of NMSS and other measures for the same construct (or other related constructs) to assess construct convergent validity (*r*). The *r*_(*s*)_ ≥ 0.50 was considered a strong correlation, 0.35 ≤ *r*_(*s*)_ < 0.5 as a moderate correlation, and *r*_(*s*)_ < 0.35 as a low correlation [31, 32]. We hypothesized that NMSS total score had a strong association with UPDRS I, SCOPA AUT total score and corresponding subscales, BDI, and PDQ-8. Besides, a low to moderate correlation was predicted between NMSS and PD severity scales (HY, SCOPA-M, and UPDRS-III), SCOPA-COG, disease duration, and LEDD, according to the previous studies [10, 33]. Internal validity was determined based on intercorrelations of NMSS domains. Known-group validity was evaluated for grouping of patients by disease duration (≤5 years and >5 years) and disease stage (H and Y)(<3 and ≥ 3). Mann–Whitney *U* test was used to assess the known-group validity.
  SPSS software version 26 was used to conduct the statistical analysis, and the *P* value less than 0.05 was considered statistically significant.

## 3. Results

### 3.1. Patients and Clinical Characteristics

A total of 186 PD patients were included in the study. The mean age of the participants was 64.46 ± 9.9 years (range 43–91). In all, 118 (63.4%) patients were men, and the mean of education was 7.63 ± 5.46 years. The mean age at the onset of PD symptoms was 58.86 ± 10.58, and the disease duration was 5.59 ± 3.99 years. The distribution of the H and Y stage was as follows: 19.9% in stage 1, 52.1% in stage 2, 23.7% in stage 3, 3.2% in stage 4, and 1.1% in stage 5. The detailed data on the clinical characteristics of patients are shown in Table 1.

### 3.2. Acceptability and Reliability

The NMSS data were computable for 99.9% of the enrolled patients (3 had missing data in the sexual domain). The acceptability and reliability of each domain of NMSS and the total score of NMSS are shown in Table 2. Considering the NMSS domains scores, only the sexual domain covered the entire possible range of scores. The mean NMSS score was 52 ± 38.5 points (range 0–234). Five patients (2.7%) stated no NMS, whereas the maximum score was 234 for one patient. The NMSS total score was higher for women (55 ± 35.2) than for men (50.3 ± 40.4), but it was not statistically significant (*p*_value_ = 0.25). All of the NMSS domains showed a floor effect; however, no ceiling effect was seen in each domain of NMSS. The most prominent floor effect was observed in the perceptual problems/hallucinations domain (81.7%), followed by the cardiovascular (56.5%), sleep/fatigue (56%), and sexual dysfunction (55.4%) domain. The total score of NMSS was free of floor and ceiling effects. The SEM values of all of the NMSS domains and total NMSS score were small (less than 1/2 SD) (Table 2).

The Cronbach's alpha coefficient of total NMSS was 0.84, consistent with good internal consistency. Considering the NMSS subscales, Cronbach's alpha coefficient ranged from 0.35 (miscellaneous) to 0.82 (mood/apathy)(Table 2). The highest item-domain correlation was seen on item 17 (“forget things or events”) (0.74), followed by item 18 (“forget to do things”) (0.71) and item 8 (“loss of interest in doing things”) (0.67). On the contrary, 4 items including item 6 (“restlessness in legs”) (0.16), item 27 (“unexplained pain”) (0.1), item 28 (“change in ability to taste/smell”) (0.15), and item 29 (“change in weight”) (0.13) showed item-domain correlation below the criterion value 0.3. Test-retest reliability demonstrated ICC 0.93 for the total NMSS score, which is excellent, and internal consistency was satisfactory. All of the NMSS domains showed ICC of more than 0.90 except for cardiovascular (0.81), gastrointestinal (0.87), and miscellaneous (0.89) domains (Table 2).

### 3.3. Validity

As hypothesized, a strong correlation was observed between the total NMSS score and UPDRS I, PDQ-8, BDI, and SCOPA-AUT. Besides, a moderate association was seen between NMSS total score and UPDRS III, and SCOPA-M, and a loose correlation was noted between NMSS and H and Y, MMSE, SCOPA COG, LEDD, and disease duration. Furthermore, a strong relationship was noted between NMSS, UPDRS II, and SCOPA-sleep, and a moderate correlation with SCOPA-PC. No significant association was noticed between NMSS total score, age, and age at the onset of the disease. Moreover, the correlation between NMSS domains and the independent scales of the related construct showed the following results: cardiac domain with SCOPA AUT cardiac domain (*r*_*s*_ = 0.52), sleep/fatigue with SCOPA-sleep (*r*_*s*_ = 0.71), sleep/fatigue with BDI (*r*_*s*_ = 0.52), mood/cognition with BDI (*r*_*s*_ = 0.7), perceptual problems/hallucinations with SCOPA-PC (*r*_*s*_ = 0.42), attention/memory with SCOPA-COG (*r*_*s*_ = −0.50), attention/memory with MMSE (*r*_*s*_ = −0.59), gastrointestinal domain with SCOPA AUT gastrointestinal domain (*r*_*s*_ = 0.65), urinary with SCOPA AUT urinary domain (*r*_*s*_ = 0.68), and sexual function with SCOPA AUT sexual domain (*r*_*s*_ = 0.75) (Table 3).

Considering known-groups validity, NMSS total score was significantly increased in the duration >5 years group compared with the duration ≤5 years group (57.6 ± 38 vs. 48 ± 38.6, *p*_value_ = 0.04). Besides, patients with H and *Y* ≥ 3 had significantly higher NMSS total scores than those with H and *Y* < 3 (67.4 ± 36.5 vs. 45.9 ± 34.8, *p*_value_ = 0.002).

Concerning internal validity, the strongest association was seen between sleep/fatigue and mood/cognition (*r*_*s*_ = 0.56), followed by sleep/fatigue and urinary (*r*_*s*_ = 0.45), and the weakest association was observed between sexual function and miscellaneous (*r*_*s*_ = 0.05), and sexual function and attention/memory (*r*_*s*_ = 0.05). The relationship between NMSS total score and NMSS subscales ranged from perceptual problems/hallucinations (*r*_*s*_ = 0.40) to mood/cognition (*r*_*s*_ = 0.79) (Supplementary Table 1).

## 4. Discussion

The Persian version of the NMSS showed satisfactory data quality, acceptability, reliability, construct, and internal validity for evaluating the burden of nonmotor symptoms in patients with PD. Considering the underestimation of nonmotor symptoms in clinical practice and the significant effect of NMS on patients' quality of life, the presence of a concise and precise questionnaire for the comprehensive and detailed assessment of the nonmotor symptoms in Iranian patients with PD is needed. To the best of our knowledge, thus far, in addition to the original version of NMSS [10], the psychometric properties of the NMSS questionnaire have been validated in Italian, Chinese, Korean, Brazilian, Arabic, and Mexican languages [34–39]. This is the first study that evaluated the clinimetric properties of the NMSS in the Iranian population.

Our study's mean total NMSS score was higher than the Italian, Korean, Chinese, and Brazilian versions of the validation [34–36] and lower than the original and Arabic versions [10, 38]. The variation in the duration of disease, severity of motor symptoms, and LEDD could explain the differences between the NMSS scores in various studies.

Similar to the previous studies [10, 34, 37, 38], the total NMSS score was free of floor and ceiling effects in our study, and compared to Cova et al. reports [34], all of the NMSS domains had floor effects and none had ceiling effects. Similar to the previous studies [10, 33], the NMSS Persian version demonstrated a skewness of 1.2, consistent with the findings of the floor effect seen in all of the NMSS domains. Our findings showed that perceptual problems/hallucinations had the highest floor effect, possibly due to the lowest prevalence of hallucinations and perceptual problems in PD, consistent with the previous suggestions [6, 10, 32, 36, 37].

The total Cronbach's alpha of the Persian version of NMSS was 0.84. Besides, three domains (mood/cognition, attention/memory, and urinary) had good internal consistency (more than 0.7), and two domains (sleep/fatigue and perceptual problems/hallucinations) showed acceptable value (more than 0.6). The rest of the NMSS domains had values lower than 0.6. The miscellaneous domain had the lowest Cronbach's alpha (0.35), consistent with the previous study [33]. Two reasons for these findings are as follows: (1) NMSS domains, particularly miscellaneous domain composed of unrelated items (pain, taste/smell, weight, and sweating); (2) the low number of items in some domains for assessing complex nonmotor symptoms.

The homogeneity of the Persian version of the NMSS domains based on item-domain correlation was acceptable in 7 domains, including cardiovascular, mood/cognition, perceptual problems/hallucinations, attention/memory, gastrointestinal, urinary, and sexual. Besides, in our study, the miscellaneous domain had the lowest item-domain correlation, which could be explained by the fact that the miscellaneous domain is composed of 4 unrelated items, similar to the previous findings [10, 33].

The SEM is regarded as a “minimally detectable change;” therefore, it can potentially represent the responsiveness of the scale [40, 41]. SEM is correlated inversely with the reproducibility index; thus, the higher ICC, the lower the SEM. Comparable to the original and Italian versions of NMSS [10, 34], our results showed that the NMSS total and all domains' SEM values were lower than half SD, implying that the Persian NMSS is a precise and potentially responsive questionnaire.

Our analysis showed a high ICC in the sexual domain (0.94), similar to the original study (0.94) [10]– and in contrast to the MartinezMartin et al. reports (0.67) [33]. The number of patients included in the test-retest (50 patients in our study and 30 in the original, versus 127 in the Martinez–Martin et al.) and the recruitment of the patients from a single center with relatively homogeneous culture (our study) versus multicenter with heterogeneous cultures and ethnicity (Martinez–Martin et al.) might illustrate this discrepancy between different studies.

Considering convergent validity, the NMSS Persian version total score showed the highest correlation with UPDRS I, followed by BDI, SCOPAAUT, PDQ-8, and SCOPA-Sleep. As a whole, NMSS total score demonstrated a moderate to high correlation with all related constructs except for SCOPA-COG and MMSE (−0.30 and −0.30), similar to the previous studies [34, 35, 37, 38]. Besides, there was a high correlation between NMSS domains and specific measures of the related construct (more than 0.50). Moreover, our results showed that NMSS total score had a moderate correlation with motor assessments, including UPDRS III and SCOPA-M, and a loose association with H and Y, similar to the original, Brazilian, and Korean versions of the validation [10, 36, 37]. However, in the Italian and Chinese versions [34, 35] of the validation, no significant correlation between NMSS and motor symptoms was observed. The severity of the motor symptoms in the enrolled patients could define this discrepancy, as the studies that included more patients in the advanced stages of the disease showed a significant correlation between NMSS and the burden of motor signs/symptoms. On the other hand, no significant association was seen between NMSS and the severity of motor assessment in the studies that included patients in the earliest stage of the disease. Consistent with the findings of the Korean version [36], the NMSS Persian version total score had a significant correlation with LEDD, implying that an increase in the dosage of dopaminergic medications can increase the severity and frequency of nonmotor symptoms. This finding could be explained by the adverse effects of the dopaminergic medications on the autonomic system (nausea, constipation, and orthostatic hypotension), behavior and perception (hallucinations, impulse control disorder, and dopamine dysregulation syndrome), and sleep (excessive daytime sleepiness, fragmented sleep, and nighttime sleep problems) [42]. Consequently, it seems that the NMSS Persian version has sufficient construct validity. Concerning the results of this study, patients with a duration of disease of more than 5 years and H and *Y* ≥ 3 had a higher NMSS score. Although the NMSS score had a loose association with H and Y and disease duration, similar to the original study [10, 33], the present study showed that NMSS has an acceptable capability in differentiating between PD patients based on the severity of disease according to H and Y and the duration of disease.

### 4.1. Limitation

One of the limitations of the present study is the low prevalence of the patients in the extreme stages of the disease, especially in the severely advanced stages (H and Y 4 and 5). The other is the lack of comparable measures to assess all items included in the NMSS, particularly in the miscellaneous domain. Moreover, our study was performed in one center and the recruited patients may not be fully representative of the total Iranian population with PD.

## 5. Conclusion

Considering the impact of nonmotor symptoms on the quality of life of patients with PD and overlooking these symptoms in clinical practice, the Persian version of NMSS provides a valid, reliable, and comprehensive instrument for the better assessment of the severity and frequency of nonmotor symptoms in Iranian patients with PD.

## Figures and Tables

**Table 1:** Score distribution of the applied measures in PD patients.

Variables	Mean (SD)	Min	Max
H&Y	2.2 (0.6)	1	5
UPDRSI	11.1 (6.6)	1	32
UPDRSII	14.4 (9.2)	0	40
UPDRSIII	36.5 (14.3)	9	83
MMSE	25.4 (4.5)	11	30
SCOPA-M	18.5 (18.1)	4	50
SCOPA-COG	27.4 (8.8)	4	38
SCOPA-Sleep	9.2 (8.2)	0	36
SCOPA-PC	1.7 (2.7)	0	18
SCOPA-AUT	10.4 (7.5)	0	39
BDI	14 (10.9)	0	45
PDQ-8	8.2 (6.4)	0	30
LEDD	666.8 (497.3)	68	2940

PD: Parkinson's disease; SD: standard deviation; H&Y: Hoehn and Yahr; UPDRS: Unified Parkinson's Disease Rating Scale; MMSE: Mini-Mental State Examination; SCOPA: Scales for Outcomes in Parkinson's Disease; M: motor; COG: cognition; PC: psychiatric complications; AUT: autonomic; BDI: Beck's Depression Inventory; PDQ-8: Parkinson's Disease Questionnaire-8 questions; LEDD: Levodopa Equivalent Daily Dose.

**Table 2:** Score distribution, acceptability, precision, and reliability of the Non-Motor Symptoms Scale in PD patients.

Variables	Mean	SD	Median	Min	Max	Skewness	Floor effect%	Ceiling effect%	SEM	Item-domaincorrelation	Cronbach's alpha	ICC
Cardiovascular	2.45	3.86	0	0	18	1.8	56.5	1.1	1.70	0.30	0.39	0.81
Sleep/fatigue	9.68	9.75	7.5	0	38	0.79	56	0.5	2.92	0.16–0.56	0.63	0.91
Mood/cognition	11.72	12.01	8	0	56	1.07	24.2	0.5	2.70	0.52–0.67	0.82	0.95
Perceptual problems/hallucinations	1.30	4.22	0	0	28	4.7	81.7	0.5	1.03	0.47–0.51	0.68	0.94
Attention/memory	5.09	6.26	4	0	29	1.44	42.5	0.5	1.25	0.48–0.74	0.8	0.96
Gastrointestinal	4.23	6.03	2	0	28	1.87	41.4	0.5	2.17	0.30–0.39	0.53	0.87
Urinary	6.54	8.28	3.5	0	33	1.35	42.5	0.5	2.48	0.48–0.58	0.71	0.91
Sexual function	4.60	6.26	0	0	24	1.17	55.4	1.6	1.53	0.41	0.56	0.94
Miscellaneous	6.36	6.85	4	0	32	0.95	37.1	0.5	2.29	0.1–0.6	0.35	0.89
NMSS total	52.01	38.54	45	0	234	1.21	2.7	0.5	10.21	—	0.84	0.93

PD: Parkinson's disease; SD: standard deviation; SEM: standard error of measurement; ICC: intra-class correlation coefficient; NMSS: Non-Motor Symptoms Scale.

**Table 3:** Construct convergent validity of the Non-Motor Symptoms Scale in PD patients.

Variables	NMSS total	Cardiovascular	Sleep/fatigue	Mood/cognition	Perceptual problems/hallucinations	Attention/memory	Gastrointestinal	Urinary	Sexual function	Miscellaneous
	*r*_*s*_^*∗*^	*r*_s_	*r*_s_	*r*_s_	*r*_s_	*r*_s_	*r*_s_	*r*_s_	*r*_s_	*r*_s_
Age	−0.06	0.03	**−0.17**	−0.04	**−0.15**	0.1	0.08	0.06	−0.06	**−0.23**
Age at onset of disease	−0.1	0.02	**−0.2**	−0.09	−0.12	0.1	0.02	0.01	−0.09	**−0.27**
Disease duration	**0.15**	0.03	0.08	0.1	0.04	−0.07	**0.14**	**0.15**	0.08	**0.14**
H&Y	**0.21**	0.1	−0.01	0.1	0.13	**0.2**	**0.2**	0.07	0.007	**0.2**
UPDRSI	**0.84**	**0.48**	**0.66**	**0.7**	**0.36**	**0.37**	**0.41**	**0.54**	**0.26**	**0.41**
UPDRSII	**0.58**	**0.38**	**0.37**	**0.4**	**0.26**	**0.26**	**0.47**	**0.32**	**0.24**	**0.4**
UPDRSIII	**0.36**	**0.23**	**0.18**	**0.2**	**0.2**	**0.25**	**0.31**	**0.2**	0.13	**0.23**
LEDD	**0.33**	**0.2**	**0.26**	0.1	**0.2**	0.01	**0.26**	**0.2**	**0.16**	**0.36**
SCOPA-AUT	**0.66**	**0.44**	**0.5**	**0.4**	**0.21**	**0.16**	**0.5**	**0.58**	**0.41**	**0.22**
SCOPA-sleep	**0.6**	**0.24**	**0.71**	**0.4**	**0.27**	0.13	**0.25**	**0.32**	**0.21**	**0.26**
SCOPA-PC	**0.41**	**0.28**	**0.26**	**0.3**	**0.42**	**0.4**	**0.2**	**0.17**	0.12	**0.17**
SCOPA-M	**0.47**	**0.35**	**0.27**	**0.3**	**0.28**	**0.22**	**0.46**	**0.24**	**0.19**	**0.26**
SCOPA-COG	**−0.3**	−0.1	**−0.2**	**−0.1**	−0.02	**−0.5**	−0.06	−0.13	0.01	−0.06
MMSE	**−0.3**	−0.1	**−0.1**	**−0.2**	−0.04	**−0.59**	0.01	−0.1	−0.02	−0.06
BDI	**0.71**	**0.38**	**0.52**	**0.7**	**0.36**	**0.26**	**0.37**	**0.38**	**0.36**	**0.31**
PDQ-8	**0.61**	**0.31**	**0.46**	**0.5**	**0.32**	**0.27**	**0.38**	**0.3**	**0.26**	**0.38**

^*∗*^ *r*_*s*_: spearman's correlation coefficient; significant correlations *P*_value_ < 0.05 are highlighted in bold. NMSS: Non-Motor Symptoms Scale; H&Y: Hoehn and Yahr; UPDRS: Unified Parkinson's disease Rating Scale; LEDD: Levodopa Equivalent Daily Dose; SCOPA: Scales for outcomes in Parkinson's Disease; AUT: autonomic; PC: psychiatric complications; M: motor; COG: cognition; MMSE: mini-Mental State Examination; BDI: Beck's Depression Inventory; PDQ-8: Parkinson's Disease Questionnaire-8 questions.

## Data Availability

The data that support the findings of this study are available from the corresponding author upon reasonable request.

## Supplementary Materials

Supplementary Materials Supplementary Table 1. Internal validity of the Non-Motor Symptoms Scale in PD patients.
