# Supplementary material for: Validation of the Non-Motor Symptoms Scale for Parkinson's Disease of Persian Version
Source: Parkinsons Dis. 2023 Jun 9;2023:1972034. doi: 10.1155/2023/1972034 (PMC10275686; doi:10.1155/2023/1972034)
Supplement: Supplementary Materials — Supplementary Table 1. Internal validity of the Non-Motor Symptoms Scale in PD patients. [file 1972034.f1.docx]

| Variables | Cardiovascular | | Sleep/fatigue | | Mood/cognition | | Perceptual problems/hallucinations | | Attention/memory | | Gastrointestinal | | Urinary | | Sexual function | | Miscellaneous | | NMSS total | |
| --- | --- | --- | --- | --- | --- | --- | --- | --- | --- | --- | --- | --- | --- | --- | --- | --- | --- | --- | --- | --- |
|  | r_s_^*^ | P^**^ | r_s_ | P | r_s_ | P | r_s_ | P | r_s_ | P | r_s_ | P | r_s_ | P | r_s_ | P | r_s_ | P | r_s_ | P |
| Cardiovascular | - | - | **0.3** | **<0.001** | **0.37** | **<0.001** | **0.21** | **0.003** | **0.17** | **0.02** | **0.3** | **<0.001** | **0.2** | **0.005** | **0.32** | **<0.001** | **0.25** | **<0.001** | **0.5** | **<0.001** |
| Sleep/fatigue | **0.3** | **<0.001** | - | - | **0.56** | **<0.001** | **0.22** | **0.003** | **0.2** | **0.005** | **0.23** | **0.002** | **0.45** | **<0.001** | **0.26** | **<0.001** | **0.26** | **<0.001** | **0.74** | **<0.001** |
| Mood/cognition | **0.37** | **<0.001** | **0.56** | **<0.001** | - | - | **0.29** | **<0.001** | **0.33** | **<0.001** | **0.33** | **<0.001** | **0.34** | **<0.001** | **0.29** | **<0.001** | **0.23** | **0.002** | **0.79** | **<0.001** |
| Perceptual problems/hallucinations | **0.21** | **0.003** | **0.22** | **0.003** | **0.29** | **<0.001** | - | - | 0.12 | 0.09 | **0.29** | **<0.001** | 0.19 | 0.008 | **0.23** | **0.001** | 0.14 | 0.06 | **0.4** | **<0.001** |
| Attention/memory | **0.17** | **0.02** | **0.2** | **0.005** | **0.33** | **<0.001** | 0.12 | 0.09 | - | - | 0.12 | 0.09 | **0.23** | **0.002** | 0.05 | 0.5 | **0.17** | **0.02** | **0.45** | **<0.001** |
| Gastrointestinal | **0.3** | **<0.001** | **0.23** | **0.002** | **0.33** | **<0.001** | **0.29** | **<0.001** | 0.12 | 0.09 | - | - | **0.34** | **<0.001** | **0.21** | **0.003** | **0.21** | **0.003** | **0.5** | **<0.001** |
| Urinary | **0.2** | **0.005** | **0.45** | **<0.001** | **0.34** | **<0.001** | **0.19** | **0.008** | **0.23** | **0.002** | **0.34** | **<0.001** | - | - | **0.23** | **0.001** | 0.13 | 0.08 | **0.62** | **<0.001** |
| Sexual function | **0.32** | **<0.001** | **0.26** | **<0.001** | **0.29** | **<0.001** | **0.23** | **0.001** | 0.05 | 0.5 | **0.21** | **0.003** | **0.23** | **0.001** | - | - | 0.05 | 0.5 | **0.45** | **<0.001** |
| Miscellaneous | **0.25** | **<0.001** | **0.26** | **<0.001** | **0.23** | **0.002** | 0.14 | 0.06 | **0.17** | **0.02** | **0.21** | **0.003** | 0.13 | 0.08 | 0.05 | 0.5 | - | - | **0.42** | **<0.001** |
| NMSS total | **0.5** | **<0.001** | **0.74** | **<0.001** | **0.79** | **<0.001** | **0.4** | **<0.001** | **0.45** | **<0.001** | **0.5** | **<0.001** | **0.62** | **<0.001** | **0.45** | **<0.001** | **0.42** | **<0.001** | - | - |

* rs : spearman’s correlation coefficient; **P: P_value_

_NMSS: Non-motor symptoms scale_
